# Corrosion Behavior of AISI 1045 Steel in Seawater in the Presence of *Flavobacterium* sp.

**DOI:** 10.3389/fmicb.2020.00303

**Published:** 2020-03-03

**Authors:** Jinyi Wu, Weixiong Zhang, Ke Chai, Aimin Yu

**Affiliations:** ^1^Key Laboratory of Tropical Biological Resources of Ministry of Education, School of Life and Pharmaceutical Sciences, School of Materials Science and Engineering, Hainan University, Haikou, China; ^2^Department of Chemistry and Biotechnology, Swinburne University of Technology, Melbourne, VIC, Australia

**Keywords:** *Flavobacterium* sp., AISI 1045 steel, corrosion, seawater, biofilm

## Abstract

A systematic comparison study was carried out to investigate the effect of *Flavobacterium* sp. on AISI 1045 steel corrosion by weight loss, fluorescence microscopy (FM), surface analysis, cell count, pH measure, electrochemical impedance spectroscopy (EIS), and polarization curves. The impedances were considerably increased by *Flavobacterium* sp. between 1 and 7 day exposure and after 30 day exposure but considerably decreased by *Flavobacterium* sp. after 15 and 21 day exposure, which were supported by the I_corr_ results and the weight loss data. Furthermore, the biofilm was formed on the coupons. The pH values were considerably decreased by *Flavobacterium* sp. after 15 and 21 day exposure. The results proved that *Flavobacterium* sp. decreased the corrosion rates between 1 and 7 day exposure and after 30 day exposure and increased the corrosion rates between 15 and 21 day exposure, which could be ascribed to the protective biofilm and the secreted corrosive acid, respectively. In addition, *Flavobacterium* sp. considerably increased the pit numbers, the maximum pit depths, and the corresponding widths and considerably decreased the E_pit_ values. Importantly, the coverage and the heterogeneity of the biofilm were positively correlated with the increases in the maximum pit depths and the corresponding widths and the decreases in the E_pit_ values by *Flavobacterium* sp. The results demonstrated that *Flavobacterium* sp. increased the pitting corrosion, which could involve the heterogeneous biofilm cover.

## Introduction

Ocean is a continuous vast body of salt water that covers more than 70% of the surface of the earth. It is home to abundant biological, mineral, and energy resources. The ocean explorations and exploitations like the ocean investigation, the ocean transportation, and the offshore oil and gas industries are now proceeding rapidly (Bhandari et al., 2015). More and more marine facilities such as ports, bridges, offshore platforms, ships, and submarine pipelines have been built. Steels are the fundamental construction materials of these marine structures (Cook, 2005). However, seawater contains numerous microorganisms and a number of corrosive matters like oxygen, hydrion, chloridion, and so on (Page, 1975; Li X. et al., 2017; Jin et al., 2019). The steels exposed in seawater are prone to corrosion due to the reactions between the steels and the corrosive matters (Melchers and Jeffrey, 2008; Popoola et al., 2013). It is worth noting that microorganisms can colonize on the surface of metals and form biofilms and change the distributions of the corrosive matters in the environment of metals (Voordouw et al., 2016; Hu et al., 2019; Dong et al., 2020). Consequently, many microorganisms can lead to the corrosion of metals, i.e., microbiologically influenced corrosion (MIC). The steels exposed in seawater are especially susceptible to MIC.

Studies have revealed many microorganisms related to MIC. Sulfate reducing bacteria (SRB) constitute an important group of corrosive bacteria (Li et al., 2018). They induce corrosion through oxidizing hydrogen at the cathodes of galvanic corrosion cells or directly oxidizing ferrum with sulfate as terminal oxidant (Dou et al., 2018; Gu et al., 2019). *Desulfovibrio vulgaris* increased the corrosion rates and the pitting corrosion of C1010 carbon steel (Chen et al., 2015), two aluminum alloys (Liu et al., 2014), copper (Jayaraman et al., 1999), and seven stainless steels (Ringas and Robinson, 1988). The mixture of *Desulfovibrio gabonensis* and *Desulfovibrio capillatus* accelerated the corrosion of SAE-1018 carbon steel (Castaneda and Benetton, 2008). *Desulfotomaculum nigrificans* considerably promoted both the general corrosion and localized corrosion of Q235 carbon steel (Liu et al., 2019). More importantly, SRB have been reported to be responsible for the corrosion deterioration of oil, power generation and marine industries, cooling water systems, etc. (Ilhan-Sungur and Çotuk, 2010; Guan et al., 2013; Li Y. et al., 2016). *Pseudomonas* is the most prevalent bacterial genus in marine environment (Yuan et al., 2008). So far, it is well known that some species of *Pseudomonas* are corrosive. *Pseudomonas* sp. enhanced the corrosion rate and the pitting corrosion of AISI 1045 steel and further reduced the tensile strength of the steel (Wu et al., 2012). *Pseudomonas aeruginosa* induced the corrosion of 2205 duplex stainless steel (Xu et al., 2017; Zhao et al., 2017), 2304 duplex stainless steel (Zhou et al., 2018), the nickel-free high nitrogen stainless steel (Li H. et al., 2016), and S32654 super austenitic stainless steel (Li H. et al., 2017). The presence of *Pseudomonas fluorescens* promoted the electrochemical reaction on single-phase Cu-Sn modern bronze, and led to pitting corrosion underneath the biofilm (Ghiara et al., 2018). Iron oxidizing bacteria (IOB) are another group of corrosive bacteria, which increase corrosion reaction by oxidizing ferrous ion to ferric ion (Chen et al., 2019). *Sphaerotilus* sp. strongly accelerated the pitting corrosion of AISI 1020 steel (Starosvetsky et al., 2001). *Acidithiobacillus ferrooxidans* in seawater accelerated the corrosion rate of C1010 steel and caused pitting corrosion (Wang H. et al., 2014). Although most studies showed that microorganisms promoted corrosion, some reports revealed the different results. Hernandez et al. (1994) reported that *Pseudomonas* sp. S9 could inhibit the corrosion of mild steel. Additionally, *Desulfovibrio* sp. inhibited the corrosion of SAE 1018 carbon steel for some time, but it became quite corrosive to the steel at longer times (Pérez et al., 2007).

*Flavobacterium* is ubiquitous in ocean (Abell and Bowman, 2005; Pesciaroli et al., 2012). Our previous work showed that when the exposure time was 365 days, the average corrosion rate of AISI 1045 steel exposed in natural seawater was about 2.1 times that of the steel exposed in sterile seawater and the content of *Flavobacterium* sp. in the corrosion products was much higher than those of the other bacteria in the corrosion products (Xiao, 2011), suggesting that *Flavobacterium* sp. might increase the steel corrosion greatly. However, very little is known about the effect of *Flavobacterium* sp. on the corrosion of metals. Moreover, AISI 1045 steel is widely used in marine structures for its low cost and excellent mechanical properties such as outstanding hardness and toughness, etc. (OrjuelaG et al., 2014). In this work, we carried out a systematic comparison of the corrosion of AISI 1045 steel exposed in sterile seawater and *Flavobacterium* sp. inoculated seawater by weight loss, fluorescence microscopy (FM), surface analysis, cell count, pH measure, electrochemical impedance spectroscopy (EIS), and polarization curves.

## Experiments

### Material

AISI 1045 steel purchased from Qiqihar Hongshun Heavy Industry Group Co. Ltd. (China) has the following composition (wt.%): 98.582 Fe, 0.499 C, 0.596 Mn, 0.230 Si, 0.028 S, 0.012 P, 0.006 Ni, 0.020 Cr, 0.001 Mo, 0.001 Nb, 0.014 Cu, 0.003 W, 0.003 Al, 0.004 V, 0.001 Ti. The carbon steel coupons with the dimensions of 50 × 25 × 3 mm, 15 × 10 × 3 mm, and Φ10 × 3 mm were used in weight loss, surface analysis, and electrochemical measurement, respectively. The coupons were sequentially abraded with 120, 400, 800, 1200, and 1500 grit *SiC* paper to obtain smooth surfaces. Then, they were degreased with acetone, rinsed with distilled water and ethanol, dried in air aseptically. Subsequently, all the coupons were kept in a desiccator before the measurement.

### Culture of *Flavobacterium* sp.

The *Flavobacterium* sp. used in this study was separated from the corrosion products of AISI 1045 steel exposed in nature seawater for 12 months. *Flavobacterium* sp. was cultured at 26°C for 2 days in 2216E medium, which contained 1 g yeast extract, 5 g peptone, and 1000 mL natural seawater. The pH of medium was adjusted to 7.8 with 1 M NaOH solution and the medium was sterilized in an autoclave at 121°C for 20 min before use; 20 mL of the bacterial culture solution was mixed with 2000 mL of sterile seawater. The mixture was cultured at 26°C for 24 h. The prepared coupons were exposed in *Flavobacterium* sp. inoculated seawater and sterile seawater at 26°C. The pH values of sterile seawater and *Flavobacterium* sp. inoculated seawater were measured by a pH-meter after 3, 7, 15, 21, and 30 day exposure.

### Weight Loss

Clarke’ s solution (antimonous oxide, 20 g; stannous chloride, 50 g; 36% hydrochloric acid, 1 L) was used to clean the coupon, after which the coupon was rinsed with distilled water and analytically pure ethanol. The weight loss was obtained after the coupon had been dried. The average corrosion rate was calculated using the following Eq. (1).

(1) V⁢(m⁢m/a)=(K×W)/(A×T×D)

where *V* is the average corrosion rate, *mm/a*; *W* is the weight loss of the coupon, g; *K* is 3.65 × 10^3^; *A* is the total area of the coupon, cm^2^; *T* is the exposure time, day; and *D* is the density of the coupon, g/cm^3^.

### Fluorescence Microscopy

Fluorescence microscopy was applied to observe the changes of the *Flavobacterium* sp. biofilm. After exposure, the coupon was rinsed with phosphate buffer saline (PBS), and stained with acridine orange (AO) for 5 min. The images were captured under a fluorescence microscope (Mshot MF41). The biofilm coverages were extracted from 20 random different images of the coupon using the software V9.0. The heterogeneity of the biofilm of the coupon was calculated using the following Eq. (2) (Wang Z. et al., 2014).

(2) $$H=\sqrt{\frac{\sum_{i=1}^{N}{\left(S_{i}-S\right)}^{2}}{N-1}}$$

where *H* is the heterogeneity of the biofilm of the coupon; *N* is the quantity of the random different images of the coupon; *S*_*i*_ is the biofilm coverage of one image,%; *S* is the average biofilm coverage of the images, i.e., the biofilm coverage of the coupon, %.

### Surface Analysis

The surfaces of the coupons were observed by scanning electron microscopy (SEM) (Hitachi S-4800) after 7, 15, and 30 day exposure. The coupons were treated according to the previously reported method (Yuan et al., 2008) with a minimum revision. Briefly, the coupons were rinsed with PBS solution twice and fixed in a 2.5 vol% PBS solution of glutaraldehyde for 8 h at 4°C, and then they were washed twice with deionized water to remove glutaraldehyde and dehydrated with 25, 50, 75, 90, 100 vol% stepwise ethanol for 10 min each. At last, they were dried in an airtight desiccator. To observe the corrosion morphology on the underlying metal surfaces, the biofilm and the corrosion products of the coupons were removed after 7, 15, and 30 day exposure, according to the above method in weight loss. The maximum pit depths and the corresponding widths were measured by 3D laser scanning confocal microscopy (Keyence VK-X250K).

### Cell Count

The living cells in *Flavobacterium* sp. inoculated seawater were counted after 3, 7, 15, 21, and 30 day exposure; 10 mL of *Flavobacterium* sp. inoculated seawater were serially diluted with sterile seawater; 100 μL solutions of the appropriate dilutions were inoculated on solid medium and incubated at 26°C for 48 h. Bacterial numbers were determined by the plate count.

### Electrochemical Analysis

A PARSTAT 2273 electrochemical workstation (Princeton Applied Research) with a three-electrode system was used for the measurements of EIS and polarization curves. The saturated calomel electrode (SCE) and the platinum electrode were employed as the reference electrode and the counter electrode, respectively. The working electrode was a coupon encapsulated in epoxy with an end surface exposed and the other end surface connected to a wire. The test solutions were sterile seawater and *Flavobacterium* sp. inoculated seawater, respectively. The test temperature was kept at 26°C. The EIS was measured at the open circuit potential (OCP) with an amplitude sinusoidal signal of 10 mV and the frequency range of 0.005–100,000 Hz. The equivalent electrical circuits (EECs) were determined using evaluator software (Zsimpwin). The sweep of polarization curves was performed with a voltage range from -1.4 to 0.4 V vs. SCE at a scan rate of 2.0 mV/s to determine the corrosion potential (E_corr_) and the corrosion current density (I_corr_).

## Results

### Average Corrosion Rates

**FIGURE 1:**
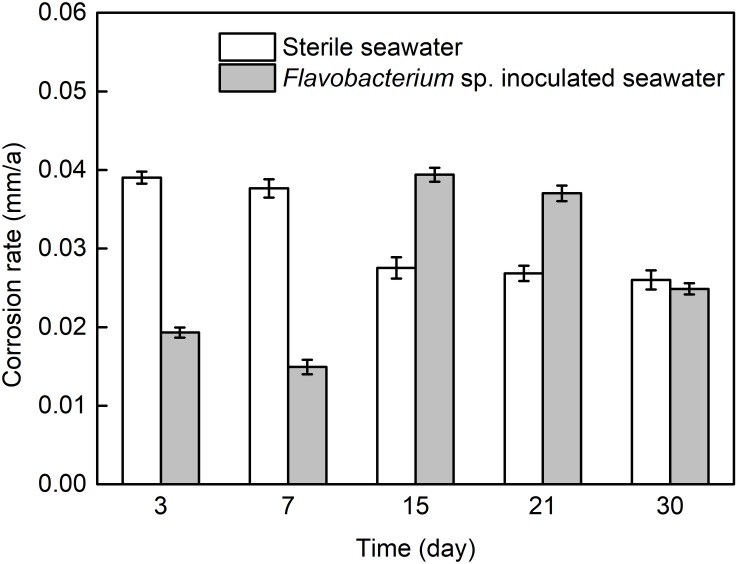
The average corrosion rates of AISI 1045 steel exposed in sterile seawater and *Flavobacterium* sp. inoculated seawater for the different times. The error bars represent the standard deviations of the six tests.

Figure 1 presents the average corrosion rates of AISI 1045 steel after 3, 7, 15, 21, and 30 day exposure in sterile seawater and *Flavobacterium* sp. inoculated seawater. The average corrosion rate in sterile seawater slightly decreased from 3 to7 day exposure and considerably decreased from 7 to 15 day exposure. At last, it slightly decreased from 15 to 21 day exposure and 21 to 30 day exposure. The average corrosion rate in *Flavobacterium* sp. inoculated seawater decreased from 3 to 7 day exposure, considerably increased from 7 to 15 day exposure and slightly decreased from 15 to 21 day exposure. Subsequently, it considerably decreased from 21 to 30 day exposure and reached a slightly lower level than that after 30 day exposure in sterile seawater. The average corrosion rates in *Flavobacterium* sp. inoculated seawater were considerably lower than the corresponding ones in sterile seawater after 3 and 7 day exposure. However, the average corrosion rates in *Flavobacterium* sp. inoculated seawater were considerably higher than the corresponding ones in sterile seawater after 15 and 21 day exposure.

### Fluorescence Microscopy

**FIGURE 2:**
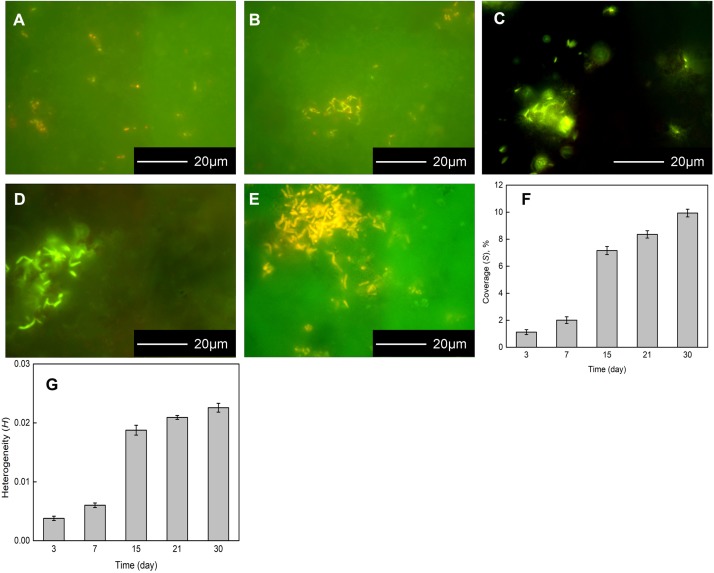
Fluorescence images of bacterial cells attaching to the coupons exposed in *Flavobacterium* sp. inoculated seawater for 3 **(A)**, 7 **(B)**, 15 **(C)**, 21 **(D)**, and 30 days **(E)** and the coverages of the biofilm **(F)** and the heterogeneities of the biofilm **(G)** after 3, 7, 15, 21, and 30 day exposure. The error bars represent the standard deviations of the six tests.

The *Flavobacterium* sp. biofilm on the coupons were presented by FM after 3, 7, 15, 21, and 30 day exposure (Figures 2A–E). Sparse *Flavobacterium* sp. cells attached to the coupon surface dispersedly and only two tiny cell clusters were distinguished after 3 day exposure (Figure 2A). After 7 day exposure, some bacterial cells clustered on the coupon surface and sparse ones distributed around (Figure 2B). With the exposure time increasing, more *Flavobacterium* sp. cells accumulated to form bigger and denser colonies, besides a small number of cells still distributed around (Figures 2C–E). Especially, the biggest and densest cell colony was detected after 30 day exposure (Figure 2E). Accordingly, the coverage and the heterogeneity of the biofilm increased with exposure time. Meanwhile, they considerably increased from 7 to 15 day exposure (Figures 2F,G).

### Surface Analysis

**FIGURE 3:**
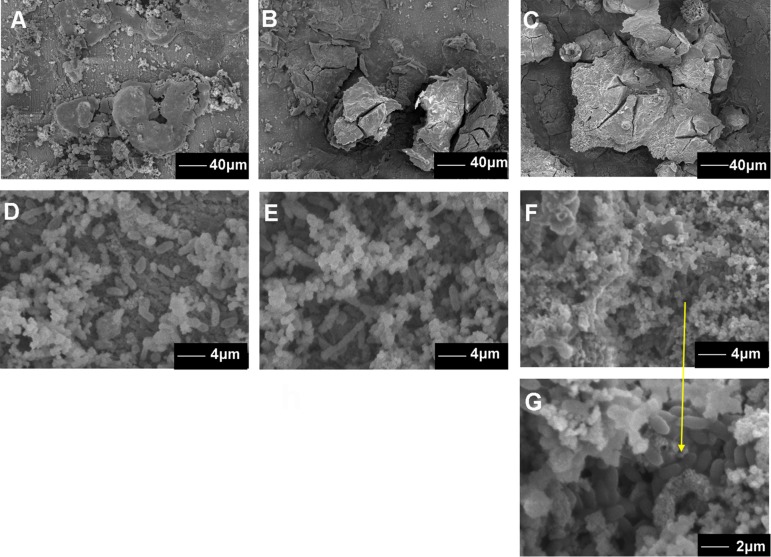
The SEM images of the surface morphology of the AISI 1045 steel coupons after 7 **(A)**, 15 **(B)**, and 30 day exposure in sterile seawater **(C)** and 7 **(D)**, 15 **(E)**, and 30 day exposure in *Flavobacterium* sp. inoculated seawater **(F,G)**.

The surface morphology of the coupons after 7, 15, and 30 day exposure showed that the corrosion products film covered the coupons exposed in sterile seawater (Figures 3A–C) and the biofilm/the corrosion products film covered the coupons exposed in *Flavobacterium* sp. inoculated seawater (Figures 3D–G). In sterile seawater, the corrosion scales were lumpy and many corrosion tubercles formed (Figures 3A–C). The amount of the corrosion products increased with exposure time (Figures 3A–C). In *Flavobacterium* sp. inoculated seawater, some *Flavobacterium* sp. cells adhered with the corrosion products to the coupon surface after 7 day exposure (Figure 3D). More and denser cells accumulated in the corrosion products on the coupons after 15 day exposure (Figure 3E). The quantity and the density of cells reached the maximum after 30 day exposure (Figures 3F,G).

**FIGURE 4:**
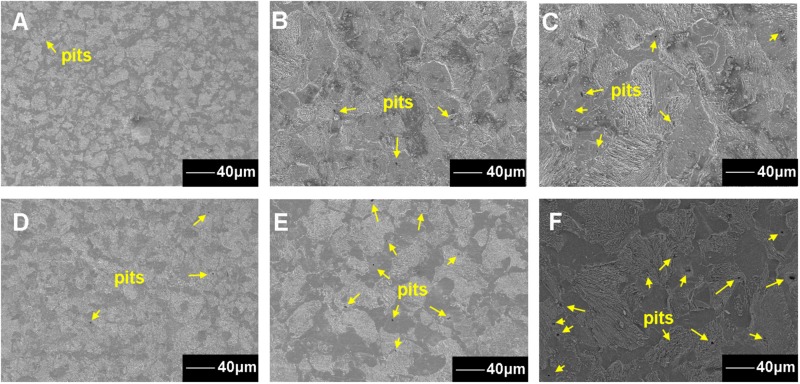
The SEM images of the metal surface corrosion morphology after the removal of the corrosion products and the biofilm from the coupons after 7 **(A)**, 15 **(B)**, and 30 day exposure in sterile seawater **(C)** and 7 **(D)**, 15 **(E)**, and 30 day exposure in *Flavobacterium* sp. inoculated seawater **(F)**.

**FIGURE 5:**
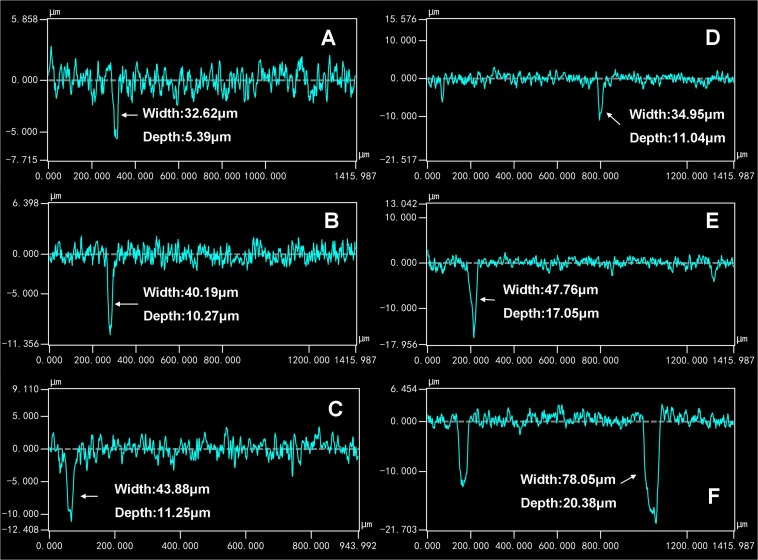
The maximum pit depths and the corresponding widths of the coupons after 7 **(A)**, 15 **(B)**, and 30 day exposure in sterile seawater **(C)** and 7 **(D)**, 15 **(E)**, and 30 day exposure in *Flavobacterium* sp. inoculated seawater **(F)**.

For the coupons exposed in sterile seawater for 7, 15, and 30 days, the pit number increased with the exposure time (Figures 4A–C). For the coupons exposed in *Flavobacterium* sp. inoculated seawater for 7, 15, and 30 days, the pit number also increased with the exposure time and the pit numbers were considerably higher than the corresponding ones of the coupons exposed in sterile seawater (Figures 4A–F). The maximum pit depths were 5.39, 10.27, and 11.25 μm with the widths of 32.62, 40.19, and 43.88 μm after 7, 15, and 30 day exposure in sterile seawater, respectively (Figures 5A–C). After 7, 15, and 30 day exposure in *Flavobacterium* sp. inoculated seawater, the maximum pit depths were 11.04, 17.05, and 20.38 μm, increasing by 5.65, 6.78, and 9.13 μm against the corresponding ones in sterile seawater, respectively (Figures 5A–F). The corresponding widths were 34.95, 47.76, and 78.05 μm, growing by 2.33, 7.57, and 34.17 μm against the corresponding ones in sterile seawater, respectively (Figures 5A–F).

### Cell Count

**FIGURE 6:**
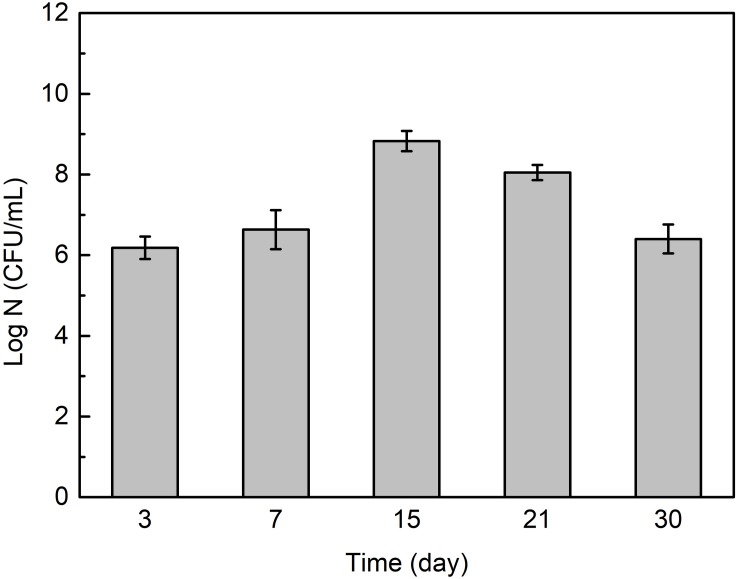
The cell numbers in *Flavobacterium* sp. inoculated seawater after the different time exposure. The error bars represent the standard deviations of the six tests.

Figure 6 shows that the cell number in *Flavobacterium* sp. inoculated seawater changed with exposure time. It slightly increased from 3 to 7 day exposure and considerably increased from 7 to 15 day exposure. It then considerably decreased from 15 to 21 day exposure and 21 to 30 day exposure.

### pH Analysis

**FIGURE 7:**
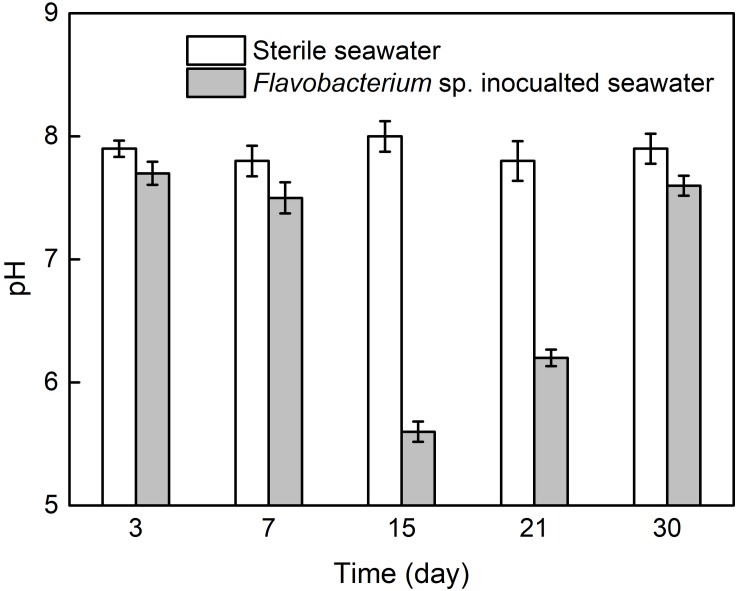
The pH values in sterile seawater and *Flavobacterium* sp. inoculated seawater after the different time exposure. The error bars represent the standard deviations of the six tests.

Figure 7 presents the pH values in the two seawaters after 3, 7, 15, 21, and 30 day exposure. The pH values in sterile seawater stayed at the similar levels around 8.0 after 3, 7, 15, 21, and 30 day exposure. In *Flavobacterium* sp. inoculated seawater, the pH values were only slightly lower than the corresponding ones in sterile seawater after 3, 7, and 30 day exposure and considerably lower than the corresponding ones in sterile seawater after 15 and 21 day exposure. In addition, the pH value in *Flavobacterium* sp. inoculated seawater slightly decreased from 3 to 7 day exposure and considerably decreased from 7 to 15 day exposure. Then it considerably increased from 15 to 21 day exposure and 21 to 30 day exposure.

### EIS Analysis

**FIGURE 8:**
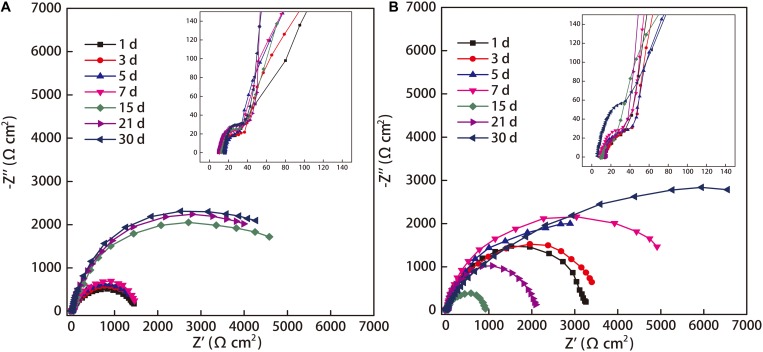
Nyquist plots of the coupons exposed in sterile seawater **(A)** and *Flavobacterium* sp. inoculated seawater **(B)**.

Electrochemical measurement is an efficient and fast method characterizing the transient electrochemical reactions occurring among metal surface, corrosion products, and biofilm (Mansfeld and Little, 1991). The EIS data acquired for the coupons exposed in the two seawaters are shown in Figure 8, in which the inset plots at high-frequency were included. As shown in the Nyquist plots (Figures 8A,B), there were two time constants present in the impedance spectra in the two seawaters. They consisted of an impedance loop at high-frequency and an impedance loop at low-frequency. In sterile seawater, the diameter of impedance loop at low-frequency slightly increased with exposure time between 1 and 7 day exposure, considerably increased from 7 to 15 day exposure and slightly increased from 15 to 21 day exposure and 21 to 30 day exposure (Figure 8A). In *Flavobacterium* sp. inoculated seawater, the diameter of impedance loop at low-frequency increased with exposure time between 1 and 7 day exposure (Figure 8B). Then it considerably decreased from 7 to 15 day exposure and reached the minimum (Figure 8B). Hereafter, it considerably increased from 15 to 21 day exposure and 21 to 30 day exposure (Figure 8B). Importantly, the diameters of impedance loops at low-frequency were considerably greater in *Flavobacterium* sp. inoculated seawater than in sterile seawater after 1, 3, 5, 7, and 30 day exposure, respectively (Figures 8A,B), but considerably smaller in *Flavobacterium* sp. inoculated seawater than in sterile seawater after 15 and 21 day exposure, respectively (Figures 8A,B). The results were consistent with the results of the average corrosion rates.

**FIGURE 9:**
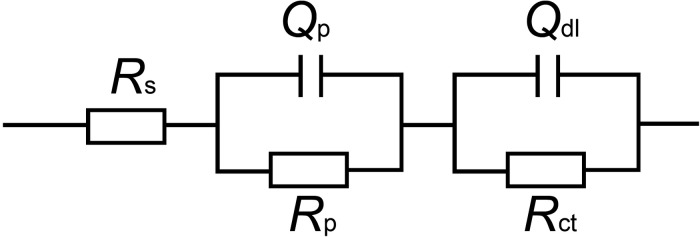
Equivalent electrical circuit used to fit the EIS data: model of R(QR)(QR) for the coupons exposed in sterile seawater and *Flavobacterium* sp. inoculated seawater. R_s_: the solution resistance, Q_p_: the constant phase element (CPE) of the corrosion products film in sterile seawater or the CPE of the biofilm/the corrosion products film in *Flavobacterium* sp. inoculated seawater, R_p_: the resistance of the corrosion products film in sterile seawater or the resistance of the biofilm/the corrosion products film in *Flavobacterium* sp. inoculated seawater, Q_dl_: the constant phase element (CPE) of the electrical double layer, R_ct_: the charge transfer resistance.

The EIS data were simulated theoretically using the EEC that had two time constants as shown in Figure 9. The goodness of the fitting was evaluated by the chi-squared error (χ^2^) between the experimental data and the fitting results. The error in the fitting was in the order of 10^–4^ in all cases (Table 1), which indicated that the EEC could be reliably used to fit the EIS data (Liu J.C. et al., 2015). The SEM observation revealed that the corrosion products film covered the coupons exposed in sterile seawater and the biofilm/the corrosion products film covered the coupons exposed in *Flavobacterium* sp. inoculated seawater. For the biofilm/the corrosion products film, *Flavobacterium* sp. cells adhered with the corrosion products to the coupon surface, indicating that the contributions of the biofilm and the corrosion products film to the EEC were indistinguishable. So the R(QR)(QR) model was fit for the coupons exposed in sterile seawater and *Flavobacterium* sp. inoculated seawater (Qu et al., 2015), in which R_s_, Q_p_, R_p_, Q_dl_, and R_ct_, respectively, stood for the solution resistance, the constant phase element (CPE) of the corrosion products film in sterile seawater or the CPE of the biofilm/the corrosion products film in *Flavobacterium* sp. inoculated seawater, the resistance of the corrosion products film in sterile seawater or the resistance of the biofilm/the corrosion products film in *Flavobacterium* sp. inoculated seawater, the CPE of the electrical double layer, and the charge transfer resistance. The fitted electrochemical parameters are listed in Table 1. The R_p_ value in sterile seawater increased from 1 to 30 day exposure. The R_ct_ value in sterile seawater slightly increased with exposure time between 1 and 7 day exposure, considerably increased from 7 to 15 day exposure, slightly increased from 15 to 21 day exposure and 21 to 30 day exposure. The R_p_ and the R_ct_ values in *Flavobacterium* sp. inoculated seawater increased with exposure time between 1 and 7 day exposure, considerably decreased from 7 to 15 day exposure and considerably increased from 15 to 21 day exposure and 21 to 30 day exposure. The R_p_ and the R_ct_ values in *Flavobacterium* sp. inoculated seawater were considerably higher than the corresponding ones in sterile seawater after 1, 3, 5, 7, and 30 day exposure. Nevertheless, the R_p_ and the R_ct_ values in *Flavobacterium* sp. inoculated seawater were considerably lower than the corresponding ones in sterile seawater after 15 and 21 day exposure. The results were in accord with the average corrosion rate results and the Nyquist data.

**TABLE 1:** Fitted parameters of EIS after the different time exposure in sterile seawater and *Flavobacterium* sp. inoculated seawater.

	**Sterile seawater**						
**Time (day)**	**1**	**3**	**5**	**7**	**15**	**21**	**30**
R_s_(Ω cm^2^)	9.34	11.62	16.15	10.36	12.65	11.89	17.14
Q_p_(F cm^–2^)	0.0008732	0.001419	0.003113	0.004149	0.004201	0.006256	0.009076
R_p_(Ω cm^2^)	82.73	93.08	100.3	176.6	218.6	256.1	279.3
Q_dl_(F cm^–2^)	0.0005302	0.003665	0.001027	0.003066	0.001453	0.001225	0.01179
R_ct_(Ω cm^2^)	1408	1475	1524	1653	4768	4886	5070
χ^2^ (10^–4^)	6.13	4.85	7.04	5.58	8.31	2.85	6.44

***Flavobacterium* sp. inoculated seawater**							

R_s_(Ω cm^2^)	18.15	9.817	9.094	8.569	10.62	9.542	7.835
Q_p_(F cm^–2^)	0.001536	0.001232	0.006864	0.004722	0.003931	0.003286	0.0007082
R_p_(Ω cm^2^)	127.7	143.9	195.3	229.7	93.4	121.4	978.9
Q_dl_(F cm^–2^)	0.0005906	0.001151	0.003206	0.002005	0.003989	0.001216	0.0005143
R_ct_(Ω cm^2^)	3118	3458	4512	4865	819.4	2031	6341
χ^2^ (10^–4^)	3.91	5.45	7.28	3.63	8.52	4.22	5.36

### Polarization Curves

**FIGURE 10:**
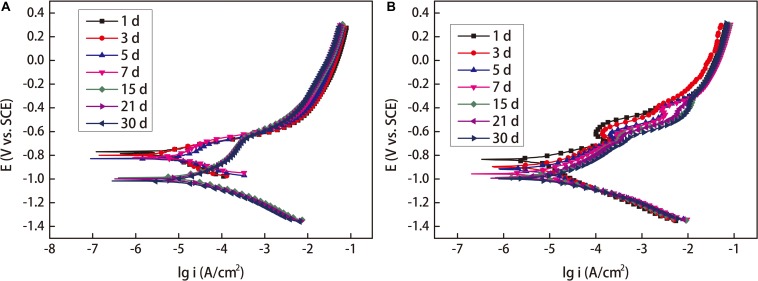
Tafel polarization curves of the coupons exposed in sterile seawater **(A)** and *Flavobacterium* sp. inoculated seawater **(B)** for the different times.

Figure 10 shows the Tafel polarization curves of the coupons exposed in sterile seawater and *Flavobacterium* sp. inoculated seawater. Tafel polarization parameters such as the corrosion potential (E_corr_), the anodic and cathodic Tafel slops (b_a_ and b_c_), the corrosion current density (I_corr_), and the pitting potential (E_pit_) extracted from the Tafel polarization curves are listed in Table 2. In sterile seawater, the corrosion current density (I_corr_) values stayed at the higher levels between 1 and 7 day exposure. Then the I_corr_ value considerably decreased from 7 to 15 day exposure and slightly decreased from 15 to 21 day exposure and 21 to 30 day exposure. In *Flavobacterium* sp. inoculated seawater, the I_corr_ value decreased with exposure time between 1 and 7 day exposure, considerably increased from 7 to 15 day exposure and considerably decreased from 15 to 21 day exposure and 21 to 30 day exposure. The I_corr_ values were considerably higher in sterile seawater than in *Flavobacterium* sp. inoculated seawater after 1, 3, 5, 7, and 30 day exposure, respectively. However, the I_corr_ values were considerably lower in sterile seawater than in *Flavobacterium* sp. inoculated seawater after 15 and 21 day exposure, respectively. The results were in agreement with the results of the average corrosion rates and the EIS. The pitting potential (E_pit_) values decreased over exposure time in the two seawaters. In addition, the E_pit_ values in *Flavobacterium* sp. inoculated seawater were considerably lower than the corresponding ones in sterile seawater between 1 and 30 day exposure. The results supported the surface analysis results.

**TABLE 2:** Parameters of Tafel polarization curves of the coupons exposed in sterile seawater and *Flavobacterium* sp. inoculated seawater for the different times.

	**Sterile seawater**				
**Time (day)**	**I_corr_ (μA/cm^2^)**	**E_corr_ (mV vs. SCE)**	**b_a_ (mV/dec)**	**b_c_ (mV/dec)**	**E_pit_ (mV vs. SCE)**
1	6.02	−770	57	−134	−300
3	6.67	−800	63	−168	−352
5	7.05	−827	253	−120	−372
7	5.55	−819	109	−126	−381
15	2.04	−989	159	−115	−421
21	1.94	−998	162	−172	−430
30	1.86	−1016	153	−116	−446

***Flavobacterium* sp. inoculated seawater**					

1	3.21	−834	92	−148	−312
3	2.92	−895	188	−132	−381
5	2.18	−914	185	−113	−460
7	1.91	−956	154	−98	−479
15	10.83	−986	310	−253	−607
21	4.01	−993	211	−153	−623
30	1.25	−998	128	−87	−650

## Discussion

The EIS is the powerful tool for detecting the different electrochemical processes at the interfaces between the electrodes and the electrolytes. The impedance loop diameter in the Nyquist plot, the R_ct_ value, and the R_p_ value are the key indicators of a corrosion rate (Liu H. et al., 2015, Batmanghelich et al., 2017; Liu et al., 2017; Song et al., 2018). The higher values of the three data represent the higher electrical resistances at the interfaces between the coupons and the electrolytes and the lower corrosion rates. In this study, the EIS data showed two time constants in the two seawaters. The time constant at high frequency corresponded to the corrosion products film formed on the coupon exposed in sterile seawater or the biofilm/the corrosion products film formed on the coupon exposed in *Flavobacterium* sp. inoculated seawater. The time constant at low frequency represented the charge transfer process in the electrical double layer, which was established at the steel and seawater interface. Many authors had found similar results. The impedance spectra of carbon steel Q235 in soil-extract solution with *Desulfovibrio desulfuricans* revealed that the time constants in the high frequency region were due to the forming of the corrosion products and the biofilm, and the time constant in the low frequency region was due to the electrical double layer (Xu et al., 2012). The EIS of 2205 duplex stainless steel corrosion by *P. aeruginosa* showed that the time constant at the higher frequency was most likely due to the biofilm/the corrosion products film, while the time constant at the lower frequency could be attributed to the electrical double layer (Xu et al., 2017). The EIS of 5052 aluminum alloy had two time constants: the one at high-frequency was associated with the formation of corrosion product in the sterile solution or the corrosion product and biofilm in *Desulfovibrio caledoniensis* media, while the other one at low-frequency was associated with the electrical double layer (Guan et al., 2017). So the two time constants at high frequency and low frequency were related to R_p_ and R_ct_, respectively.

In sterile seawater, the impedance loop diameter, the R_ct_ value, and the R_p_ value increased from 1 to 30 day exposure. Nevertheless, the average corrosion rate and the I_corr_ value decreased from 3 to 30 day exposure and from 1 to 30 day exposure, respectively. These changes illustrated that the corrosion rate of the steel exposed in sterile seawater decreased from 1 to 30 day exposure. These results could be due to the increase of the amount of the corrosion products on the coupon, which could decrease the transmission of corrosive ions and molecules to the steel surface (Li S. et al., 2016). Similarly, the resistance of the corrosion products film formed on AISI 304 stainless steel increased in the first 10 days of immersion in sterile seawater due to the increase in thickness of the corrosion products (Rodríguez et al., 2006). The corrosion product increased the impedance value of AZ31B magnesium alloy immersed in the artificial seawater (Kang et al., 2019). The increase of the R_ct_ value of the naval carbon steel BV-grade A in natural seawater was due to the increase in the thickness and/or compactness of the corrosion products layer (Belkaid et al., 2011). The pit number, the maximum pit depth, and the corresponding width results in sterile seawater showed the pitting corrosion of the steel exposed in sterile seawater, which could be attributed to the concentration cells caused by the heterogeneous corrosion products film (Fadl-allah et al., 2016).

The EIS data showed that the impedances including the impedance loop diameters, the R_ct_ values, and the R_p_ values in *Flavobacterium* sp. inoculated seawater considerably increased after 1, 3, 5, 7, and 30 day exposure and considerably decreased after 15 and 21 day exposure as compared to their counterparts in sterile seawater, which were in accord with the I_corr_ results of the polarization curves and the weight loss data. These results demonstrated that *Flavobacterium* sp. decreased the corrosion rates of the steel between 1 and 7 day exposure and after 30 day exposure and increased the corrosion rates between 15 and 21 day exposure. The FM and the SEM results revealed that *Flavobacterium* sp. formed the biofilm on the coupons. The biofilm acted as a transport barrier, blocking the diffusion of the corrosive species such as oxygen and chloridion to the steel surface (Mohanan et al., 2004; Guo et al., 2017). Even it could prevent the diffusion of the corrosion products (Qu et al., 2015). Besides, the biofilm could remove oxygen at the steel/electrolyte interface through oxygen respiration (Little and Ray, 2002). These led to the increases in the impedances and decreases in the I_corr_ values, the average corrosion rates, and the corrosion rates. Thus, the decreases of the corrosion rates by *Flavobacterium* sp. could be attributed to the corrosion inhibition by the biofilm, which were supported by previous studies. *Bacillus subtilis* increased the impedance value and decreased the corrosion rate due to the formation of the biofilm on the AZ31B magnesium alloy surface (Kang et al., 2019). The *P. aeruginosa* biofilm increased the R_ct_ values of nickel-zinc alloys, suggesting that this species inhibited corrosion on nickel-zinc surfaces (San et al., 2014). *D. desulfuricans* increased the R_ct_ value of carbon steel Q235 and decreased the corrosion rate, which was attributed to the protective ability of the biofilm (Sun et al., 2011). However, the cell count data and the pH results showed that in *Flavobacterium* sp. inoculated seawater, the cell number variations with exposure time were contrary to the pH value profiles, indicating that the acid secreted by *Flavobacterium* sp. was correlated to the stages of the cell proliferation, that is the amount of the secreted acid increased with the cell number increase and decreased with the cell number decrease due to nutrient starvation. These results were consistent with the profiles of the organic acid secretion by *B. subtilis*, in which the organic acid secreted by *B. subtilis* is associated with the reproduction of the microorganism (Qu et al., 2015). Importantly, the pH values in *Flavobacterium* sp. inoculated seawater considerably decreased relative to the corresponding ones in sterile seawater after 15 and 21 day exposure and reached the acidic values about 5.6 and 6.2. Acting as the depolarizer, the acid secreted by *Flavobacterium* sp. increased the cathodic depolarization, i.e., hydrogen evolution reaction, and decreased the deposition of the corrosion products on the steel surface through decreasing the concentration of hydroxyl radical. These caused the decreases in the impedances and increases in the I_corr_ values, the average corrosion rates, and the corrosion rates. So the increases of the corrosion rates by *Flavobacterium* sp. could be attributed to the corrosion acceleration by the acid from *Flavobacterium* sp., which were supported by the following results. The acetate produced by *D. desulfuricans* decreased the environmental pH and increased the corrosion rate of the iron (Pak et al., 2003). The organic acids such as lactic acid, fumaric acid, malic acid, and linoleic acid produced by *Trichoderma harzianum* decreased the R_ct_ value and the R_p_ value of AZ31B magnesium alloy in artificial seawater and increased the corrosion rate (Qu et al., 2017). The low pH attributed to the acid produced by the iron-oxidizing bacteria enhanced the corrosion rate of the petrol tank (Manga et al., 2012).

The surface analysis results showed that the pit numbers, the maximum pit depths, and the corresponding widths in *Flavobacterium* sp. inoculated seawater considerably increased as compared to their counterparts in sterile seawater. The polarization curves results showed that the E_pit_ values in *Flavobacterium* sp. inoculated seawater considerably decreased as compared to their counterparts in sterile seawater. These results demonstrated that *Flavobacterium* sp. increased the pitting corrosion of the steel. Moreover, the FM results showed that the coverage and the heterogeneity of the biofilm increased with exposure time. The increases in the maximum pit depth and the corresponding width and the decrease in the E_pit_ value by *Flavobacterium* sp. exhibited the similar trends to the two formers. The bacterial accumulation depended on the communication of the bacteria through the released quorum sensing signal molecules (Fuqua et al., 1994; Hamzah et al., 2013). In turn, the signal molecules were assembled by the accumulating bacteria (Waters and Bassler, 2005). As time went on, the biofilm became more massive (Bollinger et al., 2001). More importantly, the biofilm became more heterogeneous (Hentzer and Givskov, 2003). The aggressive ions and molecules diffused more difficultly to the steel surface areas coated by the biofilm than to the uncoated areas, which formed the concentration cells. Furthermore, the oxygen consumption by the biofilm aggravated the differences in oxygen concentration between the coated and the uncoated areas. The higher the coverage and the heterogeneity of the biofilm the greater the concentration differences of the corrosive substances between the coated and the uncoated areas, which served as the anodes and the cathodes of the concentration cells, respectively (Zarasvand and Rai, 2014). The anodic dissolution led to the pitting corrosion. So the pitting corrosion by *Flavobacterium* sp. could be attributed to the heterogeneous biofilm cover. Similarly, the increases in thickness and heterogeneity of the *P. aeruginosa* biofilm caused the more severe pitting corrosion of mild steel (Abdolahi et al., 2015). The increases of the *Desulfovibrio alaskensis* biofilm porosity and heterogeneity led to the increases of the pitting corrosion of St37-2 (S235-JR) carbon steel (Wikieł et al., 2014). The heterogeneous biofilm of M11 and S8-5 SRB resulted in the gradients of pH, sulfate, and chloride and the pitting corrosion of mild steel (Xu et al., 2002).

## Conclusion

In this study, AISI 1045 steel corrosion by *Flavobacterium* sp. was explored by weight loss, FM, surface analysis, cell count, pH measure, EIS, and polarization curves. *Flavobacterium* sp. considerably increased the impedances after 1, 3, 5, 7, and 30 day exposure and considerably decreased the impedances after 15 and 21 day exposure, which were in agreement with the I_corr_ results and the weight loss data. Additionally, the *Flavobacterium* sp. biofilm formed on the coupons. *Flavobacterium* sp. considerably decreased the pH values after 15 and 21 day exposure. These results demonstrated that *Flavobacterium* sp. decreased the corrosion rates between 1 and 7 day exposure and after 30 day exposure and increased the corrosion rates between 15 and 21 day exposure, which was associated with the corrosion inhibition by the biofilm and the corrosion acceleration by the secreted acid, respectively. In addition, *Flavobacterium* sp. considerably increased the pit numbers, the maximum pit depths, and the corresponding widths and considerably decreased the E_pit_ values. Importantly, together with the increases in the maximum pit depth and the corresponding width and the decrease in the E_pit_ value by *Flavobacterium* sp., the coverage and the heterogeneity of the biofilm increased with exposure time. The results demonstrated that *Flavobacterium* sp. increased the pitting corrosion, which could be attributed to the heterogeneous biofilm cover.

## Data Availability Statement

All datasets generated for this study are included in the article/supplementary material.

## Author Contributions

JW and WZ performed the experiments. JW, WZ, and KC analyzed the data, and wrote the manuscript. JW and KC designed the experiments and modified the manuscript. AY modified the manuscript.

## Conflict of Interest

The authors declare that the research was conducted in the absence of any commercial or financial relationships that could be construed as a potential conflict of interest.

## References

[B1] Abdolahi A. Hamzah E. Ibrahim Z. Hashim S. (2015). Localised corrosion of mild steel in presence of *Pseudomonas aeruginosa* biofilm. *Corros. Eng. Sci. Technol.* 50 538–546. 10.1179/1743278215Y.0000000003

[B2] Abell G. C. J. Bowman J. P. (2005). Ecological and biogeographic relationships of class *Flavobacteria* in the Southern Ocean. *FEMS Microbiol. Ecol.* 51 265–277. 10.1016/j.femsec.2004.09.001 16329875

[B3] Batmanghelich F. Li L. Seo Y. (2017). Influence of multispecies biofilms of *Pseudomonas aeruginosa* and *Desulfovibrio vulgaris* on the corrosion of cast iron. *Corros. Sci.* 121 94–104. 10.1016/j.corsci.2017.03.008

[B4] Belkaid S. Ladjouzi M. A. Hamdani S. (2011). Effect of biofilm on naval steel corrosion in natural seawater. *J. Solid State Electrochem.* 15 525–537. 10.1007/s10008-010-1118-5

[B5] Bhandari J. Khan F. Abbassi R. Garaniya V. Ojeda R. (2015). Modelling of pitting corrosion in marine and offshore steel structures-A technical review. *J. Loss Prev. Process Ind.* 37 39–62. 10.1016/j.jlp.2015.06.008

[B6] Bollinger N. Hassett D. J. Iglewski B. H. Costerton J. W. McDermott T. R. (2001). Gene expression in *Pseudomonas aeruginosa*: evidence of iron override effects on quorum sensing and biofilm-specific gene regulation. *J. Bacteriol.* 183 1990–1996. 10.1128/JB.183.6.1990-1996.2001 11222597 PMC95094

[B7] Castaneda H. Benetton X. D. (2008). SRB-biofilm influence in active corrosion sites formed at the steel-electrolyte interface when exposed to artificial seawater conditions. *Corros. Sci.* 50 1169–1183. 10.1016/j.corsci.2007.11.032

[B8] Chen S. Liu G. Zhang D. (2019). Corrosion of Q235 carbon steel in seawater containing *Mariprofundus ferrooxydans* and *Thalassospira* sp. *Front. Microbiol.* 10:936. 10.3389/fmicb.2019.00936 31134004 PMC6517491

[B9] Chen Y. Tang Q. Senko J. Cheng G. Newby B. M. Z. Castaneda H. (2015). Long-term survival of *Desulfovibrio vulgaris* on carbon steel and associated pitting corrosion. *Corros. Sci.* 90 89–100. 10.1016/j.corsci.2014.09.016

[B10] Cook D. C. (2005). Spectroscopic identification of protective and non-protective corrosion coatings on steel structures in marine environments. *Corros. Sci.* 47 2550–2570. 10.1016/j.corsci.2004.10.018

[B11] Dong Y. Lekbach Y. Li Z. Xu D. Abed S. E. Koraichi S. I. (2020). Microbiologically influenced corrosion of 304L stainless steel caused by an alga associated bacterium *Halomonas titanicae*. *J. Mater. Sci. Technol.* 37 200–206. 10.1016/j.jmst.2019.06.023

[B12] Dou W. Jia R. Jin P. Liu J. Chen S. Gu T. (2018). Investigation of the mechanism and characteristics of copper corrosion by sulfate reducing bacteria. *Corros. Sci.* 144 237–248. 10.1016/j.corsci.2018.08.055

[B13] Fadl-allah S. A. Montaser A. A. El-Rab S. M. G. (2016). Biocorrosion control of electroless Ni-Zn-P coating based on carbon steel by the *pseudomonas aeruginosa* biofilm. *Int. J. Electrochem. Sci.* 11 5490–5506. 10.20964/2016.07.96

[B14] Fuqua W. C. Winans S. C. Greenberg E. P. (1994). Quorum sensing in bacteria: the LuxR-LuxI family of cell density-responsive transcriptional regulators. *J. Bacteriol.* 176 269–275. 10.1128/jb.176.2.269-275.1994 8288518 PMC205046

[B15] Ghiara G. Spotorno R. Trasatti S. P. Cristiani P. (2018). Effect of *Pseudomonas fluorescens* on the electrochemical behaviour of a single-phase Cu-Sn modern bronze. *Corros. Sci.* 139 227–234. 10.1016/j.corsci.2018.05.009

[B16] Gu T. Jia R. Unsal T. Xu D. (2019). Toward a better understanding of microbiologically influenced corrosion caused by sulfate reducing bacteria. *J. Mater. Sci. Technol.* 35 631–636. 10.1016/j.jmst.2018.10.026

[B17] Guan F. Zhai X. Duan J. Zhang J. Li K. Hou B. (2017). Influence of sulfate-reducing bacteria on the corrosion behavior of 5052 aluminum alloy. *Surf. Coat. Technol.* 316 171–179. 10.1016/j.surfcoat.2017.02.057

[B18] Guan J. Xia L. P. Wang L. Y. Liu J. F. Gu J. D. Mu B. Z. (2013). Diversity and distribution of sulfate-reducing bacteria in four petroleum reservoirs detected by using 16S rRNA and dsrAB genes. *Int. Biodeterior. Biodegrad.* 76 58–66. 10.1016/j.ibiod.2012.06.021

[B19] Guo Z. Liu T. Cheng Y. F. Guo N. Yin Y. (2017). Adhesion of *Bacillus subtilis* and *Pseudoalteromonas lipolytica* to steel in a seawater environment and their effects on corrosion. *Colloids Surf. B* 157 157–165. 10.1016/j.colsurfb.2017.05.045 28586728

[B20] Hamzah E. Hussain M. Z. Ibrahim Z. Abdolahi A. (2013). Influence of *Pseudomonas aeruginosa* bacteria on corrosion resistance of 304 stainless steel. *Corros. Eng. Sci. Technol.* 48 116–120. 10.1179/1743278212Y.0000000052

[B21] Hentzer M. Givskov M. (2003). Pharmacological inhibition of quorum sensing for the treatment of chronic bacterial infections. *J. Clin. Invest.* 112 1300–1307. 10.1172/JCI20074 14597754 PMC228474

[B22] Hernandez G. Kucera V. Thierry D. Pedersen A. Hermansson M. (1994). Corrosion inhibition of steel by bacteria. *Corrosion* 50 603–608. 10.5006/1.3293532

[B23] Hu Y. Xiao K. Zhang D. Yi P. Xiong R. Dong C. (2019). Corrosion acceleration of printed circuit boards with an immersion silver layer exposed to *Bacillus cereus* in an aerobic medium. *Front. Microbiol.* 10:1493. 10.3389/fmicb.2019.01493 31312193 PMC6614184

[B24] Ilhan-Sungur E. Çotuk A. (2010). Microbial corrosion of galvanized steel in a simulated recirculating cooling tower system. *Corros. Sci.* 52 161–171. 10.1016/j.corsci.2009.08.049

[B25] Jayaraman A. Ornek D. Duarte D. A. Lee C. C. Mansfeld F. B. Wood T. K. (1999). Axenic aerobic biofilms inhibit corrosion of copper and aluminum. *Appl. Microbiol. Biotechnol.* 52 787–790. 10.1007/s00253005159 10616712

[B26] Jin Y. Li Z. Zhou E. Lekbach Y. Xu D. Jiang S. (2019). Sharing riboflavin as an electron shuttle enhances the corrosivity of a mixed consortium of *Shewanella oneidensis* and *Bacillus licheniformis* against 316L stainless steel. *Electrochim. Acta* 316 93–104. 10.1016/j.electacta.2019.05.094

[B27] Kang Y. Li L. Li S. Zhou X. Xia K. Liu C. (2019). Temporary inhibition of the corrosion of AZ31B magnesium alloy by formation of *Bacillus subtilis* biofilm in artificial seawater. *Materials* 12:523. 10.3390/ma12030523 30744166 PMC6384576

[B28] Li H. Yang C. Zhou E. Yang C. Feng H. Jiang Z. (2017). Microbiologically influenced corrosion behavior of S32654 super austenitic stainless steel in the presence of marine *Pseudomonas aeruginosa* biofilm. *J. Mater. Sci. Technol.* 33 1596–1603. 10.1016/j.jmst.2017.03.002

[B29] Li H. Zhou E. Ren Y. Zhang D. Xu D. Yang C. (2016). Investigation of microbiologically influenced corrosion of high nitrogen nickel-free stainless steel by *Pseudomonas aeruginosa*. *Corros. Sci.* 111 811–821. 10.1016/j.corsci.2016.06.017

[B30] Li S. Bacco A. C. Birbilis N. Cong H. (2016). Passivation and potential fluctuation of Mg alloy AZ31B in alkaline environments. *Corros. Sci.* 112 596–610. 10.1016/j.corsci.2016.08.022

[B31] Li X. Duan J. Xiao H. Li Y. Liu H. Guan F. (2017). Analysis of bacterial community composition of corroded steel immersed in Sanya and Xiamen seawaters in China via method of illumina MiSeq sequencing. *Front. Microbiol.* 8:1737. 10.3389/fmicb.2017.01737 28955315 PMC5601074

[B32] Li Y. Jia R. Al-Mahamedh H. H. Xu D. Gu T. (2016). Enhanced biocide mitigation of field biofilm consortia by a mixture of D-amino acids. *Front. Microbiol.* 7:896. 10.3389/fmicb.2016.00896 27379039 PMC4904036

[B33] Li Y. Xu D. Chen C. Li X. Jia R. Zhang D. (2018). Anaerobic microbiologically influenced corrosion mechanisms interpreted using bioenergetics and bioelectrochemistry: a review. *J. Mater. Sci. Technol.* 34 1713–1718. 10.1016/j.jmst.2018.02.023

[B34] Little B. Ray R. (2002). A perspective on corrosion inhibition by biofilms. *Corrosion* 58 424–428. 10.5006/1.3277632

[B35] Liu F. Zhang J. Sun C. Yu Z. Hou B. (2014). The corrosion of two aluminium sacrificial anode alloys in SRB-containing sea mud. *Corros. Sci.* 83 375–381. 10.1016/j.corsci.2014.03.003

[B36] Liu H. Fu C. Gu T. Zhang G. Lv Y. Wang H. (2015). Corrosion behavior of carbon steel in the presence of sulfate reducing bacteria and iron oxidizing bacteria cultured in oilfield produced water. *Corros. Sci.* 100 484–495. 10.1016/j.corsci.2015.08.023

[B37] Liu H. Gu T. Asif M. Zhang G. Liu H. (2017). The corrosion behavior and mechanism of carbon steel induced by extracellular polymeric substances of iron-oxidizing bacteria. *Corros. Sci.* 114 102–111. 10.1016/j.corsci.2016.10.025

[B38] Liu H. Meng G. Li W. Gu T. Liu H. (2019). Microbiologically influenced corrosion of carbon steel beneath a deposit in CO2-saturated formation water containing *Desulfotomaculum nigrificans*. *Front. Microbiol.* 10:1298. 10.3389/fmicb.2019.01298 31244809 PMC6581712

[B39] Liu J. C. Park S. Nagao S. Nogi M. Koga H. Ma J. S. (2015). The role of Zn precipitates and Cl- anions in pitting corrosion of Sn–Zn solder alloys. *Corros. Sci.* 92 263–271. 10.1016/j.corsci.2014.12.014

[B40] Manga S. S. Oyeleke S. B. Ibrahim A. D. Aliero A. A. Bagudo A. I. (2012). Influence of bacteria associated with corrosion of metals. *CJMB* 6 19–25.

[B41] Mansfeld F. Little B. (1991). A technical review of electrochemical techniques applied to microbiologically influenced corrosion. *Corros. Sci.* 32 247–272. 10.1016/0010-938X(91)90072-W

[B42] Melchers R. E. Jeffrey R. (2008). Modeling of long-term corrosion loss and pitting for chromium-bearing and stainless steels in seawater. *Corrosion* 64 143–154. 10.5006/1.3280683

[B43] Mohanan S. Maruthamuthu S. Venkatachari G. Palaniswamy N. Raghavan M. (2004). Corrosion inhibition by freshwater biofilm on 316 stainless steel. *Bull. Electrochem.* 20 129–132. 10.1016/j.jpowsour.2003.09.031

[B44] OrjuelaG A. Rincón R. Olaya J. J. (2014). Corrosion resistance of niobium carbide coatings produced on AISI 1045 steel via thermo-reactive diffusion deposition. *Surf. Coat. Technol.* 259 667–675. 10.1016/j.surfcoat.2014.10.012

[B45] Page C. L. (1975). Mechanism of corrosion protection in reinforced concrete marine structures. *Nature* 258 514–515. 10.1038/258514a0

[B46] Pak K. R. Lee H. J. Lee H. K. Kim Y. K. Oh Y. S. Choi S. C. (2003). Involvement of organic acid during corrosion of iron coupon by *Desulfovibrio desulfuricans*. *J. Microbiol. Biotechnol.* 13 937–941.

[B47] Pérez E. J. Cabrera-Sierra R. González I. Ramírez-Vives F. (2007). Influence of *Desulfovibrio* sp. *biofilm on SAE* 1018 carbon steel corrosion in synthetic marine medium. *Corros. Sci.* 49 3580–3597. 10.1016/j.corsci.2007.03.034

[B48] Pesciaroli C. Cupini F. Selbmann L. Barghini P. Fenice M. (2012). Temperature preferences of bacteria isolated from seawater collected in Kandalaksha Bay, White Sea, Russia. *Polar Biol.* 35 435–445. 10.1007/s00300-011-1091-1

[B49] Popoola L. T. Grema A. S. Latinwo G. K. Gutti B. Balogun A. S. (2013). Corrosion problems during oil and gas production and its mitigation. *Int. J. Ind. Chem.* 4:35. 10.1186/2228-5547-4-35 30229628

[B50] Qu Q. He Y. Wang L. Xu H. Li L. Chen Y. (2015). Corrosion behavior of cold rolled steel in artificial seawater in the presence of *Bacillus subtilis* C2. *Corros. Sci.* 91 321–329. 10.1016/j.corsci.2014.11.032

[B51] Qu Q. Li S. Li L. Zuo L. Ran X. Qu Y. (2017). Adsorption and corrosion behaviour of *Trichoderma harzianum* for AZ31B magnesium alloy in artificial seawater. *Corros. Sci.* 118 12–23. 10.1016/j.corsci.2017.01.005

[B52] Ringas C. Robinson F. P. A. (1988). Corrosion of stainless steel by sulfate-reducing bacteria—electrochemical techniques. *Corrosion* 44 386–396. 10.5006/1.3583953

[B53] Rodríguez J. S. Hernández F. S. González J. G. (2006). Comparative study of the behaviour of AISI 304 SS in a natural seawater hopper, in sterile media and with SRB using electrochemical techniques and SEM. *Corros. Sci.* 48 1265–1278. 10.1016/j.corsci.2005.04.007

[B54] San N. O. Nazır H. Dönmez G. (2014). Microbially influenced corrosion and inhibition of nickel-zinc and nickel-copper coatings by *Pseudomonas aeruginosa*. *Corros. Sci.* 79 177–183. 10.1016/j.corsci.2013.11.004

[B55] Song W. Chen X. He C. Li X. Liu C. (2018). Microbial corrosion of 2205 duplex stainless steel in oilfield-produced water. *Int. J. Electrochem. Sci.* 13 675–689. 10.20964/2018.01.54

[B56] Starosvetsky D. Armon R. Yahalom J. Starosvetsky J. (2001). Pitting corrosion of carbon steel caused by iron bacteria. *Int. Biodeterior. Biodegrad.* 47 79–87. 10.1016/S0964-8305(99)00081-5 28715664

[B57] Sun C. Xu J. Wang F. (2011). Interaction of sulfate-reducing bacteria and carbon steel Q 235 in biofilm. *Ind. Eng. Chem. Res.* 50 12797–12806. 10.1021/ie200952y

[B58] Voordouw G. Menon P. Pinnock T. Sharma M. Shen Y. Venturelli A. (2016). Use of homogeneously-sized carbon steel ball bearings to study microbially-influenced corrosion in oil field samples. *Front. Microbiol.* 7:351. 10.3389/fmicb.2016.00351 27047467 PMC4805590

[B59] Wang H. Ju L. K. Castaneda H. Cheng G. Newby B. M. Z. (2014). Corrosion of carbon steel C1010 in the presence of iron oxidizing bacteria *Acidithiobacillus ferrooxidans*. *Corros. Sci.* 89 250–257. 10.1016/j.corsci.2014.09.005

[B60] Wang Z. Gao J. Ai T. Jiang W. Zhao P. (2014). Quantitative evaluation of carbon fiber dispersion in cement based composites. *Constr. Build. Mater.* 68 26–30. 10.1016/j.conbuildmat.2014.06.035

[B61] Waters C. M. Bassler B. L. (2005). Quorum sensing: cell-to-cell communication in bacteria. *Annu. Rev. Cell Dev. Biol.* 21 319–346. 10.1146/annurev.cellbio.21.012704.131001 16212498

[B62] Wikieł A. J. Datsenko I. Vera M. Sand W. (2014). Impact of *Desulfovibrio alaskensis* biofilms on corrosion behaviour of carbon steel in marine environment. *Bioelectrochemistry* 97 52–60. 10.1016/j.bioelechem.2013.09.008 24238898

[B63] Wu J. Y. Xiao W. L. Yang Y. H. Cao Y. Chai K. (2012). Influence of *Pseudomonas* on corrosion and mechanical properties of carbon steel in sea water. *Corros. Eng. Sci. Technol.* 47 91–95. 10.1179/1743278211y.0000000031

[B64] Xiao W. L. (2011). *Effect of Microbial Corrosion Process on the Mechanical Properties of Carbon Steel in Tropical Seawater Condition.* Master’s thesis, Hainan University, Haikou.

[B65] Xu D. Xia J. Zhou E. Zhang D. Li H. Yang C. (2017). Accelerated corrosion of 2205 duplex stainless steel caused by marine aerobic *Pseudomonas aeruginosa* biofilm. *Bioelectrochemistry* 113 1–8. 10.1016/j.bioelechem.2016.08.001 27578208

[B66] Xu J. Sun C. Yan M. Wang F. (2012). Effects of sulfate reducing bacteria on corrosion of carbon steel Q235 in soil-extract solution. *Int. J. Electrochem. Sci.* 7 11281–11296.

[B67] Xu L. C. Chan K. Y. Fang H. H. (2002). Application of atomic force microscopy in the study of microbiologically influenced corrosion. *Mater. Charact.* 48 195–203. 10.1016/S1044-5803(02)00239-5

[B68] Yuan S. J. Pehkonen S. O. Ting Y. P. Kang E. T. Neoh K. G. (2008). Corrosion behavior of type 304 stainless steel in a simulated seawater-based medium in the presence and absence of aerobic *Pseudomonas* NCIMB 2021 bacteria. *Ind. Eng. Chem. Res.* 47 3008–3020. 10.1021/ie071536x

[B69] Zarasvand K. A. Rai V. R. (2014). Microorganisms: induction and inhibition of corrosion in metals. *Int. Biodeterior. Biodegrad.* 87 66–74. 10.1016/j.ibiod.2013.10.023

[B70] Zhao Y. Zhou E. Liu Y. Liao S. Li Z. Xu D. (2017). Comparison of different electrochemical techniques for continuous monitoring of the microbiologically influenced corrosion of 2205 duplex stainless steel by marine *Pseudomonas aeruginosa* biofilm. *Corros. Sci.* 126 142–151. 10.1016/j.corsci.2017.06.024

[B71] Zhou E. Li H. Yang C. Wang J. Xu D. Zhang D. (2018). Accelerated corrosion of 2304 duplex stainless steel by marine *Pseudomonas aeruginosa* biofilm. *Int. Biodeterior. Biodegrad.* 127 1–9. 10.1016/j.ibiod.2017.11.003

